# Evaluation of Potential Probiotic Properties of *Limosilactobacillus fermentum* Derived from Piglet Feces and Influence on the Healthy and *E. coli*-Challenged Porcine Intestine

**DOI:** 10.3390/microorganisms11041055

**Published:** 2023-04-18

**Authors:** Mengqi Qian, Xinchen Zhou, Tingting Xu, Meng Li, Zhiren Yang, Xinyan Han

**Affiliations:** 1 Hainan Institute, Zhejiang University, Yazhou Bay Sci-Tech City, Sanya 572000, China12117031@zju.edu.cn (M.L.);; 2 College of Animal Science, Zhejiang University, Hangzhou 310058, China

**Keywords:** *Limosilactobacillus fermentum*, porcine intestinal organoid, apicobasal polarity, intestinal epithelia, Enterotoxigenic *Escherichia coli* K88, Wnt/β-catenin pathway

## Abstract

In this work, we evaluated the probiotic properties of *Limosilactobacillus fermentum* strains (FL1, FL2, FL3, FL4) isolated from feces of healthy piglets. The in vitro auto-aggregation, hydrophobicity, biofilm-forming capacity, survival in the gastrointestinal tract, antimicrobial activity and anti-oxidation capacity were evaluated. Four strains were resistant to simulated gastrointestinal conditions, including low pH, pepsin, trypsin and bile salts. They also maintained strong self-aggregation and cell surface hydrophobicity. *Limosilactobacillus fermentum* FL4, which had the strongest adhesion ability and antimicrobial effect on Enterotoxigenic *Escherichia coli* K88 (ETEC K88), was then tested in porcine intestinal organoid models. The in vitro experiments in basal-out and apical-out organoids demonstrated that *L. fermentum* FL4 adhered to the apical surfaces more efficiently than basolateral surfaces, had the ability to activate the Wnt/β-catenin pathway to protect the mucosal barrier integrity, stimulated the proliferation and differentiation of the intestinal epithelium, and repaired ETEC K88-induced damage. Moreover, *L. fermentum* FL4 inhibited inflammatory responses induced by ETEC K88 through the reduced expression of pro-inflammatory cytokines (TNF-α, IL-1β and IFN-γ) and higher levels of anti-inflammatory cytokines (TGF-β and IL-10). These results show that *L. fermentum* FL4 isolated from feces of healthy Tunchang piglets has the potential to be used as an anti-inflammatory probiotic and for mitigation of intestinal damage in piglets.

## 1. Introduction

Many microorganisms are considered to be probiotics that have beneficial effects on their hosts. Lactic acid bacteria (LAB) have been shown to be effective probiotics in swine production [1,2,3]. LAB consist of *Aerococcus, Carnobacterium, Enterococcus, Lactobacillus, Lactococcus, Leuconostoc, Oenococcus, Pediococcus, Streptococcus, Tetragenococcus, Vagococcus,* and *Weissella* [4]. Huang et al. [5] showed that the LAB count in native pig feces was much higher than in wild pig feces. Additionally, Wang et al. [6] indicated the strains of *Lactobacillus plantarum* ZLP001 isolated from the gastrointestinal tracts of healthy pigs modulated their gut microbiota. Since the natural microbiota stabilized in the intestine easily and propagated to a specific species rapidly [7], the isolates from homologous hosts themselves would be more effective probiotics than isolates from other sources. *Limosilactobacillus fermentum* is the one widely presented in nature, often isolated from natural fermented plants [8], bread [9], dairy products [10], breast milk [11], saliva [12], or human and animal feces [13,14,15]. The inherent characteristics of *L. fermentum* accelerate the improvement and development of probiotic preparations. However, *L. fermentum* is understudied compared with other species in the genus *Lactobacillus*. Existing research has proposed the application of *L. fermentum* in improving gastrointestinal disorders, colorectal cancer (CRC) and liver diseases [16,17].

Probiotics can improve intestinal health by maintaining intestinal barrier function, participating in the immune response and preventing pathogen invasions [18]. Probiotics usually adjust the expression of pro-inflammatory and anti-inflammatory cytokines to regulate the inflammatory response [19]. Probiotics inhibit excessive NF-κB-induced or TLR4-mediated pro-inflammatory cytokine production by IECs and significantly increase IL-10 secretion [14,20]. Research has revealed that the fermented milk prepared by *L. fermentum* could maintain the immune homeostasis of Th1 and Th2. Moreover, the ability of anti-*Escherichia coli* in aged mice was improved [21]. A previous study has also found that *L. fermentum* could maintain the barrier integrity when the host was suffering from the invasion of *E. coli* [22]. In addition, *Lactobacillus* produces organic acids, hydrogen peroxide (H_2_O_2_) and bacteriocins that can help protect the host from a variety of pathogens [23,24].

To study the interaction of probiotics and the intestine, researchers have investigated different experimental models in vivo or in vitro, such as Caco-2 cells [25]. With the advancement of stem cell culture, the establishment of in vitro 3D tissues called organoids has developed rapidly and been widely applied in basic research, drug discovery and regenerative medicine [26]. Intestinal organoids containing ISCs can proliferate and differentiate into all intestinal epithelial cell lineages, including absorptive, goblet, Paneth and tuft cells [27]. ISCs control epithelial cell homeostasis and regeneration, especially in response to mucosal injury and inflammation. Further, the Wnt/β-catenin signaling pathway is indispensable in modulating ISCs in crypts for stem cell expansion and crypt formation [28,29].

The aim of this work was to confirm the probiotic properties of four *Limosilactobacillus fermentum* (FL1, FL2, FL3, FL4) strains isolated from healthy Tunchang piglet feces. Then, the *L. fermentum* with the best comprehensive properties would be selected. The basal-out organoids and apical-out organoids were established to explore the effects of *L. fermentum* on the proliferation and differentiation of intestinal stem cells (ISCs) under regular and injured conditions. Ultimately, we hope that the new potential strain can be further studied in in vivo experiments and used as a commercial probiotic additive in swine production with inherent characteristics.

## 2. Materials and Methods

### 2.1. Isolation and Identification of Bacteria

This experiment was approved by the Animal Care and Use Committee of Zhejiang University [permit number SYXK 20120178]. Four *Limosilactobacillus fermentum* (FL1, FL2, FL3, FL4) strains isolated from healthy piglet feces were used in this study. The rectal contents of healthy Tunchang piglets (Tunchang, China) were collected from the anus with sterilized cotton swabs, which were stored in sterile 10 mL centrifuge tubes and immediately transferred to the laboratory. In a sterile environment, sterile water was added to form sample suspensions. The suspensions were diluted in gradient concentrations and then inoculated evenly on DeMan-Rogosa-Sharpe (MRS) agar. All of them were incubated in an anaerobic chamber (ternary gas mixture, 10% carbon dioxide, 10% hydrogen, 80% nitrogen) at 37 °C for 48 h. After manual streaking three times, single colonies were picked to be identified by 16S rDNA sequencing.

Bacterial DNA extraction was performed using a kit (TSINGKE DNA Extraction Kit, Tsingke Biotechnology, Beijing, China). Primers 27F (AGAGTTTGATCCTGGCTCAG, 1500 bp) and 1492R (TACGGCTACCTTGTTACGACTT, 1500 bp) and Tsingke 1 × TSE101 gold medal mix (Tsingke Biotechnology, China) were used to amplify the 16S rDNA gene. Cycling conditions were as follows: Initial activation at 98 °C for 2 min; 35 denaturation cycles at 98 °C for 10 s, annealing at 50 °C for 10 s, and extension at 72 °C for 15 s. The final cycle was at 72 °C for 5 min (Tsingke Biotechnology Co., Ltd.). The purified amplicon was sent to Tsingke Biotechnology (Wuhan, China) for sequencing. Sequences were aligned using MEGA 11 software and compared by BLAST to representative sequences in GenBank. 

Enterotoxigenic *Escherichia coli* K88 (ETEC K88) strain was purchased from China Veterinary Drug Administration (Beijing, China).

### 2.2. Bacterial Cultures

All *Limosilactobacillus fermentum* strains were preserved with 50% glycerol at −80 °C. Before using in experiments, cultures were activated twice in MRS medium at 37 °C in an anaerobic chamber for 24 h. 

Enterotoxigenic Escherichia coli (ETEC) K88 strain was activated twice in LB medium at 37 °C for 12 h in a shaker.

### 2.3. In Vitro Probiotic Property Tests

The probiotic properties of these strains were tested using the following assays:

Referring to Sornsenee et al. [30] with small adjustments, the cultures were centrifuged at 200× *g* for 10 min and washed with sterile PBS (pH = 6.5) (Applygen, Beijing, China) at least twice. Then, the bacterial precipitations were resuspended in PBS with similar absorbance (A_0_ = 0.60 ± 0.02) at 600 nm. The suspensions were placed in a chamber at 37 °C and the absorbance (A_t_) of the supernatant was measured at different culture times (1 h, 2 h, 3 h, 4 h, 24 h). The auto-aggregation ability was calculated as follows:

Auto-aggregation (%) = (1 − A_t_/A_0_) × 100

According to Vlkova et al. [31], measuring the adhesion to Hydrocarbons, the bacterial suspensions were obtained the same as above. The suspensions (2 mL) and toluene (2 mL) were vortex-mixed for 2 min, then incubated at 37 °C for 15 min. The phases were separated, and the aqueous phase was measured at 600 nm (A). The hydrophobicity was calculated as follows:

Hydrophobicity (%) = (1 − A/A_0_) × 100

Ten wells in a sterile 96-well plate were filled with 200 µL bacterial suspension [32]. Negative control wells had only medium added. The 96-well plate was incubated in an anaerobic chamber at 37 °C for 24 h. Then, suspensions were removed from all wells and the wells were washed three times. Fixation, 99% methanol, 15 min; Staining, 2% Hucker crystal violet, 2 min; Dissolving, 33% (*v*/*v*) glacial acetic acid. The absorbance of every well was measured at 630 nm (A).

The 24 h-cultures were inoculated into MRS medium (pH = 2.0, pH = 3.0) at 3% (*v*/*v*) inoculation, then incubated at 37 °C for 3 h. Samples were taken at 0 h and 3 h and counted on MRS agar.

Acid tolerance (%) = [(Log10 CFU/mL (3 h))/(Log10 CFU/mL (0 h))] × 100

The cultures were centrifuged at 6000 rpm for 10 min and washed with sterile PBS (pH = 6.5) at least twice. Then, the bacterial precipitations were resuspended in sterile saline with similar absorbance (A_600_ = 0.60 ± 0.02). The suspensions (1 mL) were added into artificial gastric juice (9 mL). The artificial gastric juice was prepared by adding pepsin (Macklin, Shanghai, China) in 0.2% (*w*/*v*) sterile saline at a concentration of 3.5 g/L, and the pH was adjusted to 2.0 with HCl. Samples were taken at 0 h and 3 h and counted on MRS agar. The suspensions above (1 mL) were mixed with artificial intestinal juice (9 mL). The artificial intestinal juice was prepared by using 22 g/L NaHCO_3_, 2 g/L trypsin (Macklin, China), 3 g/L bile salt (Solarbio, Beijing, China), and 4.4 g/L NaCl, and the pH was adjusted to 8.0 with NaOH. Artificial gastric and intestinal juices were filtered through a 0.22 μm sterile filter membrane. Samples were taken at 0 h and 3 h and counted on MRS agar. The bacterial survival rate was calculated as follows:

Gastric survival rate (%) = [Log10 CFU/mL (3 h))/(Log10 CFU/mL (0 h))] × 100

Intestinal survival rate (%) = [(Log10 CFU/mL (3 h))/(Log10 CFU/mL (0 h))] × 100

Agar diffusion method was used to detect the antimicrobial activity of *L. fermentum* strains against ETEC K88 strain. 10 mL LB medium containing 20 μL ETEC K88 was poured into sterile petri dish and dried in the clean bench. The sterilized oxford cup was evenly placed on the solidified medium with sterile tweezers. The ends of the tweezers were tapped slightly to ensure the oxford cup was in close contact with the medium. In the oxford cup, 50 μL *L. fermentum* was added, with PBS as negative control and neomycin sulfate as positive control. After the sample was added, all plates were transferred to an incubator and cultured at 37 °C for about 18 h. The inhibition zone diameters were measured with calipers.

Total antioxidant capacity (TAC) of supernatants was measured using the total antioxidant capacity assay kit-FRAP method (Jiancheng, China). Total antioxidant capacity was represented by the concentration of FeSO_4_ standard solution (mmol/gprot).

### 2.4. Porcine Intestinal Organoid Cultivation

The porcine intestinal organoids were kindly provided by Professor Xiaoliang Li at Zhejiang University. The cryopreserved organoids were placed in a 37 °C water bath for 2 min and then contents were transferred into DMEM/F-12 (ThermoFisher, Waltham, MA, USA) with 1% BSA (Biofroxx, Hesse, Germany). The suspensions were centrifuged at 200× *g* for 5 min to obtain the cell pellets, which were further resuspended in 100 μL complete organoid culture medium (STEMCELL Technologies, Vancouver, BC, Canada) and seeded in 100 μL Matrigel (BD Bioscience, Franklin, NJ, USA) on a prewarmed 24-well culture plate (Corning, Coring, NY, USA). Matrigel was incubated for 15 min at 37 °C to polymerize. Finally, 500 μL complete organoid culture medium supplemented with 1% antibiotics-antimycotic (10,000 IU/mL Penicillin + 10,000 μg/mL Streptomycin + 25 μg/mL Amphotericin B) (Beyotime, Shanghai, China) was added to every well. Intestinal organoids were cultured at 37 °C in a 5% CO_2_ chamber. Culture medium was replaced every 2 days and the organoids were passaged every 5 or 7 days based on the growth of organoids. By dissociating the organoids with gentle cell dissociation reagent (GCDR) (STEMCELL Technologies, Vancouver, BC, Canada) on a shaker at 37 °C for 15 min, cells were obtained for seeding in fresh Matrigel. All the experiments were started after at least two passages.

### 2.5. Establishment of Intestinal Apical-Out Organoids

Suspension culture of intestinal organoids was established as described in [33] with modifications. After passaging, organoids were cultured in Matrigel for 24 h, then the medium containing approximately 200 organoids was aspirated from the wells. The Matrigel in each well was broken up using 500 μL of ice-cold 5 mM EDTA/PBS (Gibco, Waltham, MA, USA; Servicebio, Wuhan, China). The solution from every 3 wells was transferred into a 15 mL sterile tube containing 10 mL of ice-cold 5 mM EDTA/PBS, which further dissolved the Matrigel on a shaker for 1 h at 4 °C. The organoid precipitates were collected by centrifugation at 200× *g* for 5 min and then washed with 5 mL DMEM (Gibco, USA). They were centrifuged again as above, the pellets were resuspended in complete organoid culture medium and cultured in a 24-well ultra-low-attachment plate. The apical-out organoids were cultured in suspension in a 5% CO_2_ incubator at 37 °C. Culture medium was replaced every 3 days.

### 2.6. Treatment of Intestinal Organoids

Before the experiment, the bacterial concentration was determined by measuring the optical density at 600 nm. The cells were harvested by centrifugation (1200× *g*, 10 min), washed twice with 1× sterile PBS, resuspended in DMEM/F12 without antibiotics (final concentration 2 × 10^6^ CFU/mL). 500 μL DMEM/F12 was added to each well to meet the requirement of 10^6^ CFU bacteria per well.

The organoids were cultured for 5 days. To detect whether the *L. fermentum* selected from in vitro probiotic property tests had a repairing effect on damage organoids, organoids and *L. fermentum* (10^6^ CFU) were co-cultured in advance for 24 h. Then, the organoids were treated with ETEC K88 (10^6^ CFU) for 1 h to induce injury. The samples were collected for further experiments at mRNA and protein levels. To verify that the *L. fermentum* had no negative effects on organoids, organoid samples were collected separately after the 24 h co-culture of organoids and *L. fermentum* (10^6^ CFU). To observe the damage caused by ETEC K88 to organoids, organoids were co-cultured with ETEC K88 (10^6^ CFU) for 1 h [33,34].

The growth conditions of organoids were observed under the microscope and images were obtained from different experimental treatments.

### 2.7. Epithelial Barrier Integrity

Apical-out organoids were pelleted in a 2 mL tube and resuspended in a solution of 4 kDa FITC-Dextran (diluted in DMEM-phenol red-free). The suspension was cultured at room temperature for 5 min, then the 4 kDa FITC-Dextran (Sigma-Aldrich, USA) was aspirated, leaving only 10 μL at the bottom. A circle with a diameter of 2cm was drawn on the slide using a Hydrophobic Barrier Pen (PAP pen) to create a well-like region, preventing the samples from flowing randomly. Then, the 10 μL solution was transferred to the center of the circle. To build a 3D-space for keeping the organoid shape, vacuum grease was applied to the four corners of the coverslip with gentle pressing and ultimately played a role in fixation. The slides were immediately observed with a laser confocal microscope (IX81-FV1000, Olympus, Shinjuku, Japan) and the fluorescence images were obtained using FV10-ASW software (version 4.2).

### 2.8. Immunofluorescence Staining

All samples were fixed in 4% paraformaldehyde for 30 min at room temperature, then centrifuged and washed with D-PBS (Servicebio, China) three times (5 min/time). Organoids were permeabilized with 0.5% Triton X-100 (Servicebio, China) and sealed with 3% BSA solution. The primary antibody solution (1:100–1:1000 dilution) (anti-Wnt3a mouse antibody, Thermo Fisher Scientific, Waltham, MA, USA; anti-villin mouse antibody, anti-Ki67 mouse antibody, anti-β-catenin mouse antibody, anti-ZO-1 mouse antibody, Servicebio, China; anti-Lgr5 mouse antibody, Origene technologies, Rockville, MD, USA) were added and the mixture was incubated overnight at 4 °C, then centrifuged and washed with D-PBS. The organoid pellets were resuspended in secondary antibody solution and phalloidin staining solution (1:500 dilution) (iFluor^TM^ 594 Conjugated Goat anti-mouse IgG Antibody, Huabio, Hangzhou, China), and incubated for 2 h at room temperature without light. 10 μL DAPI (2 μg/mL, Servicebio, China) staining solution was added and they were incubated for 10 min at room temperature without light. After the centrifuging and washing, organoids were resuspended in 10 μL Vectashield mounting medium (Servicebio, China). The 10 μL solution was transferred to the center of the PAP pen circle and vacuum grease was applied to corners of the coverslip with gentle pressing. The slides were immediately observed under a laser confocal microscope and the fluorescence images were obtained using FV10-ASW software.

### 2.9. qRT-PCR

Basal-out organoids and apical-out organoids were collected after experiments. Total RNA was extracted from intestinal organoids and *L. fermentum* (FL4) strain by Trizol assay. RNA was further reverse-transcribed into cDNA using PrimeScript RT Master Mix (Perfect Real Time) (Takara) following the manufacturer’s instructions. All cDNA were analyzed on LightCycler 480 Real-time PCR System with the TB green Premix Ex Taq (Tli RNaseH Plus, Takara) and specific primer (Table 1). Reaction conditions: 95 °C, 5 s; 40 cycles of 95 °C, 5 s and 60 °C, 30 s; 95 °C, 5 s; 60 °C, 1 min; 95 °C, 5 s; 50 °C, 30 s. GAPDH was used as endogenous gene for porcine intestinal organoids, while phenyl alanyl synthase (*pheS*) was used as control gene for *L. fermentum*. Assays were performed in triplicate, and the relative expression of target gene was analyzed based on the 2^−ΔΔCt^ method. 

### 2.10. Metagenomic Sequencing Analysis

Total genomic DNA was extracted from feces samples using the E.Z.N.A.^®^ Soil DNA Kit (Omega Bio-tek, Norcross, GA, USA) according to the manufacturer’s instructions. Concentration and purity of extracted DNA was determined with TBS-380 and NanoDrop2000, respectively. DNA extract quality was checked on 1% agarose gel.

DNA extract was fragmented to an average size of about 400 bp using Covaris M220 (Gene Company Limited, Hong Kong, China) for paired-end library construction. Paired-end library was constructed using NEXTFLEX Rapid DNA-Seq (Bioo Scientific, Austin, TX, USA). Adapters containing the full complement of sequencing primer hybridization sites were ligated to the blunt end of fragments. Paired-end sequencing was performed on Illumina Novaseq 6000 (Illumina Inc., San Diego, CA, USA) at Majorbio Bio-Pharm Technology Co., Ltd. (Shanghai, China) using NovaSeq Reagent Kits according to the manufacturer’s instructions (www.illumina.com, accessed on 13 November 2020). Sequence data associated with this project are still being analyzed and are not yet public.

At the outset of analyzing metagenomic sequencing data, thorough splitting and cutting was needed to ensure the raw data was of high quality. The next step was assembly, which involved merging overlapping metagenomic reads into longer contiguous DNA contigs. The assembly software MEGAHIT (version 11), based on the principle of the De-Brujin graph, was used. According to the overlapping relationship between kmers, the De-Brujin graph was constructed to obtain contigs. Contigs of more than 800 bp were screened for statistics and subsequent analysis.

Open reading frames (ORFs) from each assembled contig were predicted using Prodigal/MetaGene (http://metagene.cb.k.u-tokyo.ac.jp/, accessed on 5 October 2006, version 1.15.1). The predicted ORFs with a length ≥ 100 bp were retrieved and translated into amino acid sequences using the NCBI translation table. A non-redundant gene catalog was constructed using CD-HIT (http://www.bioinformatics.org/cd-hit/, accessed on 1 September 2009, version 4.6.1) with 90% sequence identity and 90% coverage. High-quality reads were aligned to the non-redundant gene catalogs to calculate gene abundance with 95% identity using SOAPaligner (http://soap.genomics.org.cn/, accessed on 1 March 2008, version 2.21).

Representative sequences of non-redundant gene catalog were aligned to NR database with an e-value cutoff of 1 × 10^−5^ using Diamond (http://ab.inf.uni-tuebingen.de/software/diamond, accessed on 17 November 2014, version 0.8.35) for taxonomic annotations. Cluster of orthologous groups of proteins (COG) annotation for the representative sequences was performed using Diamond (http://ab.inf.uni-tuebingen.de/software/diamond, accessed on 17 November 2014, version 0.8.35) against eggNOG database with an e-value cutoff of 1 × 10^−5^. The KEGG annotation was conducted using Diamond (http://ab.inf.uni-tuebingen.de/software/diamond, accessed on 17 November 2014, version 0.8.35) against the Kyoto Encyclopedia of Genes and Genomes database (http://www.genome.jp/keeg, accessed on 17 August 2022) with an e-value cutoff of 1 × 10^−5^.

### 2.11. Statistical Analysis

The data from experiments were expressed as the mean ± SD. One-way ANOVA was evaluated to determine significant differences among multiple groups, followed by Bonferroni multiple comparisons or Dunnett’s T3 multiple comparisons (SPSS 26.0). Significant differences between two groups were analyzed by the t-test (SPSS 26.0) and data among three groups were evaluated by one-way ANOVA. *p* < 0.05 and *p* < 0.001 were considered statistically significant. 

## 3. Results

### 3.1. Isolation and Identification of Limosilactobacillus fermentum Strains

**Figure 1:**
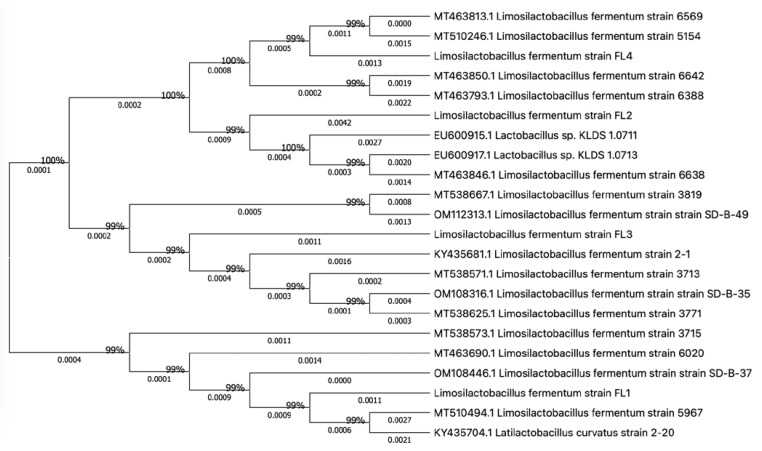
Phylogenetic tree of strains based on 16R rRNA sequence.

Through metagenomic sequencing (Supplementary Figure S1), it was found that the abundance of *Lactobacillus* and *Limosilactobacillus fermentum* was significantly higher in Tunchang pigs (TC) than in Duroc × Landrace × Yorkshire (DLY). We isolated and purified four LAB species from healthy Tunchang piglet feces after incubating under anaerobic conditions at 37 °C for 48 h. The strains were Gram-positive and rod-shaped, which was consistent with the morphological characteristics of *Lactobacillus* (Supplementary Figure S1). As shown in Figure 1, all four isolates (FL1, FL2, FL3, FL4) were identified as *Limosilactobacillus fermentum* by genotype identification and showed 99% homology to the same strains. The conservation and homology of the isolated strains were observed by multiple sequence alignment. MEGA11 (molecular evolutionary genetics analysis) software was used to perform unambiguous alignment of the 16S rRNA gene sequences of the four bacteria identified in this study to construct a phylogenetic tree. Different color codes are used for each nucleotide to show the homology and differences in the obtained sequences.

### 3.2. Tolerance of Limosilactobacillus fermentum to Gastrointestinal Tract Conditions

Following the FAO/WHO guidelines (Guidelines for the Evaluation of Probiotics in Food (2002) World Health Organization), probiotic bacteria must be safe and tolerant of gastrointestinal tract (GIT) conditions (including low pH, pepsin, pancreatin, and bile salt) The situations at pH 2 and 3 were examined in this study. The tolerance of *L. fermentum* to low pH is illustrated in Table 2. All strains showed higher viability when incubated within 3 h in MRS medium at pH 2 and 3, although the survival at pH 2 decreased after 3 h. Four *L. fermentum* strains except *L. fermentum* FL2 had a survival rate over 90% when cultured at pH 3, while FL2 had good viability at pH 2, second only to *L. fermentum* FL4. The experiment to simulate gastric conditions included the acid resistance tests in order to investigate the combined effects of low pH and pepsin. Similarly, four *L. fermentum* all survived in the simulated gastric juice (pH 2.0) after 3 h, among whom FL4 had the highest survival rate of 59.95%, followed by FL2. 

Simulation of intestinal conditions investigated the effects of bile salt and pancreatin on the growth of bacteria, and the bacterial viability under the influence of the simulated intestinal juice is presented in Table 2. After being processed by artificial gastric juice for 3 h, *L. fermentum* was also tolerant of the simulated intestinal juice and showed good survival rates. After 3 h incubation, FL4 showed the highest survival (73.57%), followed by *L. fermentum* FL3 (70.83%). According to the results above, the strain FL4 had good resistance to low pH and the gastrointestinal tract environment.

### 3.3. Adhesion Ability between Different Limosilactobacillus fermentum Strains 

**Figure 2:**
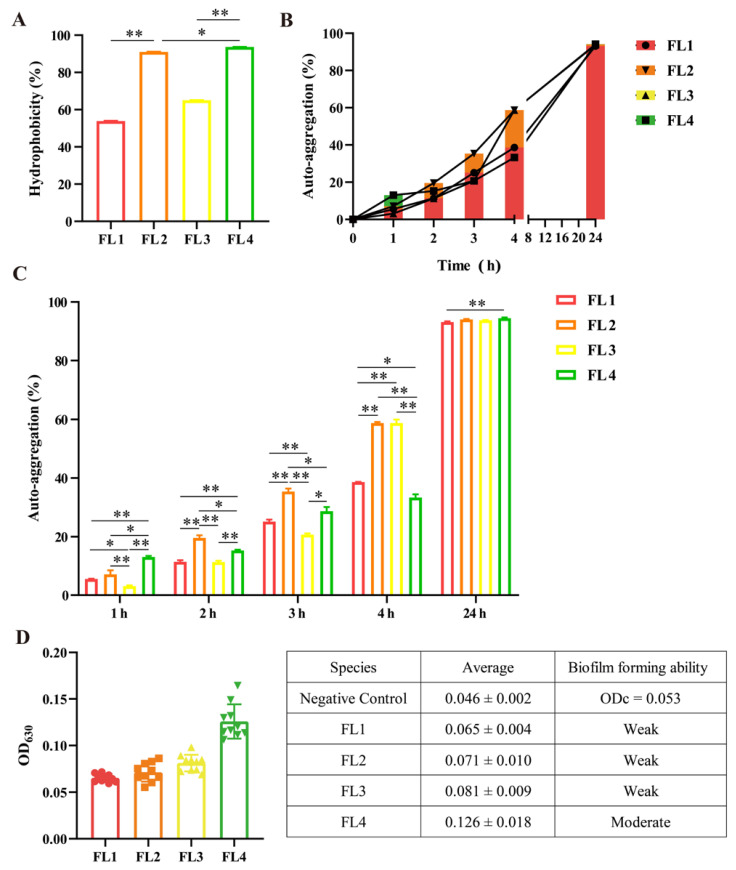
The adhesion abilities of four *L. fermentum* strains. (**A**) The cell surface hydrophobicity of isolated strains. (**B**) The trend graph of auto-aggregation ability by time. (**C**) Comparison of auto-aggregation of four *L. fermentum* strains at different times (1, 2, 3, 4 and 24 h). (**D**) The quantitative analysis of biofilm-forming capacity. Quantitative detection of biofilms: ODc = Negative Control (average) + 3 × SD. A < ODc, do not have the ability to form biofilms; ODc < A ≤ 2ODc, weak biofilm-forming ability; 2ODc < A ≤ 4ODc, moderate biofilm-forming ability; A ≥ 4ODc, strong biofilm-forming ability. Data were expressed as the mean ± SD (n = 10). * *p* < 0.05, ** *p* < 0.01.

Can a probiotic bacterium effectively exert its probiotic effect based on the capacity to adhere to the intestinal mucosal surface? The cell surface hydrophobicity, auto-aggregation and biofilm-forming capacity are positively correlated with adhesion ability, and can be considered to be indicators of the adhesion level of probiotics [35]. The four *L. fermentum* strains showed different degrees of hydrophobicity, varying from 53.90% to 90.16%. The *L. fermentum* FL4 strain showed the highest hydrophobicity, followed by *L. fermentum* FL2, showing hydrophobicity of 86.67% (Figure 2A). De souza et al. reported cell surface hydrophobicity as an important physicochemical characteristic that could facilitate the first contact between probiotics and intestinal epithelial cells [9,42]. Furthermore, the hydrophobicity also affected the auto-aggregation of bacteria [43]. Corresponding to the hydrophobicity results, the auto-aggregation test presented similar results, where FL4 was at the highest level and FL2 was second. In addition, there was an upward trend in the degree of auto-aggregation of *L. fermentum* over time. FL4 showed the fastest speed of auto-aggregation (13.07%) in just one hour, but FL2 (58.74) and FL3 (58.71%) presented higher degrees of auto-aggregation after 4 h culture (Figure 2B,C). Aggregation is an important feature for biofilm formation, so we further quantified the biofilm-forming ability of *L. fermentum*. From Figure 2D, the biofilm-forming ability of FL4 was 2.34 at the middle level but the highest of the four *L. fermentum* strains, as the other three strains were at a weak level. 

### 3.4. Antimicrobial and Anti-Oxidation Assays 

**Figure 3:**
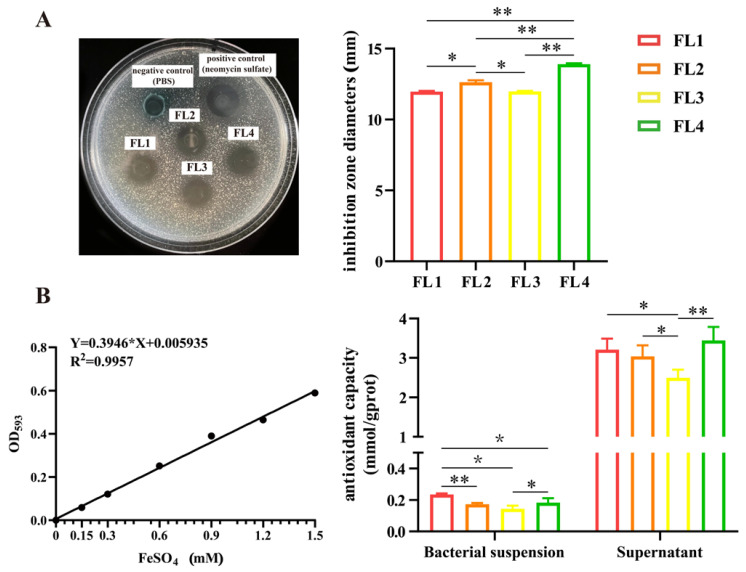
The antimicrobial and anti-oxidation ability of *L. fermentum* strains. (**A**) The zone of inhibition of *L. fermentum* against ETEC K88. (**B**) The standard curve of FeSO_4_ and the antioxidant capacity of *L. fermentum*, including its supernatant and bacterial suspension. * *p* < 0.05, ** *p* < 0.01.

Antimicrobial activity is an important characteristic for probiotics because the antimicrobial substances they produce can antagonize the invasion of harmful bacteria. The antimicrobial effect of *L. fermentum* on ETEC K88 was explored using its supernatant and suspension. The results in Figure 3A reveal that the supernatant of *L. fermentum* had inhibitory effects on ETEC K88; among all the strains, FL4 showed the largest-diameter zone of inhibition (13.91 mm). The presence of organic acids, hydrogen peroxide and bacteriocins in the supernatant lead to the inhibitory effects [35]. We measured the pH of four bacterial solutions in advance, and they were all around 4, creating a low-pH environment. 

Previous studies indicated that LAB had the antioxidant capacity to clean the reactive oxygen species (ROS) in the intestine and further keep the stable state of ROS in vivo [44]. *L. fermentum* produced a large amount of glutathiones that were natural antioxidants; in this study, total antioxidant capacity was represented by the concentration of FeSO_4_ standard solution. The standard curve and the results shown in Figure 3B show that antioxidant capacity was mainly in the supernatant rather than the bacterial suspension. The antioxidant capacity of the bacterial suspension ranged from only 0.14 to 0.24 mmol/gprot, while the supernatant of FL4 had the strongest antioxidant capacity (3.45 mmol/gprot), followed by FL1 (3.21 mmol/gprot) and FL2 (3.04 mmol/gprot). In general, the four *L. fermentum* strains all demonstrated good anti-oxidation performance and have the potential to be antioxidants.

### 3.5. L. fermentum FL4 Influenced the Proliferation of Basal-Out Intestinal Organoids

**Figure 4:**
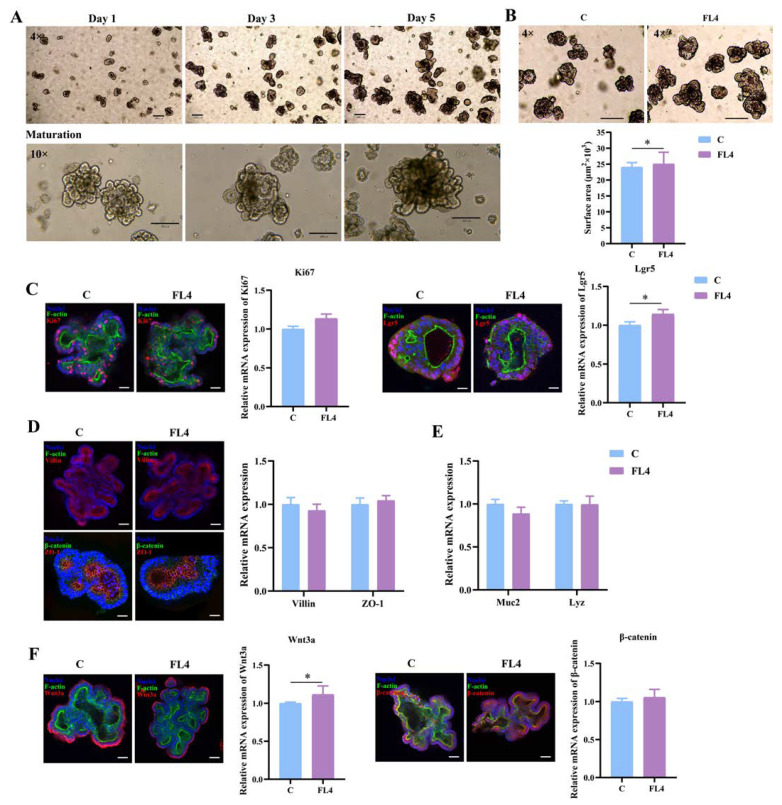
*L. fermentum* FL4 influenced the proliferation of basal-out intestinal organoids under physiological conditions. (**A**) Resuscitation of cryopreserved intestinal organoids that were passaged at least twice. Morphology of mature intestinal organoids for formal tests. 4×, scale bars, 200 μm; 10×, scale bars, 200 μm. (**B**) Organoids were treated with or without *L. fermentum* FL4 (10^6^ CFU/well) for 24 h. The surface area of organoids was calculated. 4×, scale bars, 200 μm; n = 6. (**C**,**D**) Confocal images of organoid staining with Nuclei (blue), F-actin (green), Ki67 and Lgr5 (red); scale bars, 20 μm. RT-qPCR analysis of the Ki67 and Lgr5 in organoids treated with/without FL4; n = 3. (**E**) Confocal images of organoid staining with villin and ZO-1 (red); scale bars, 20 μm. RT-qPCR analysis of the villin and ZO-1 in organoids treated with/without FL4; n = 3. (**E**) RT-qPCR analysis of the Muc2, lysozyme (Lyz) in organoids treated with/without FL4; n = 3. (**F**) Confocal images of organoid staining with Wnt3a and β-catenin (red); scale bars, 20 μm. RT-qPCR analysis of the Wnt3a and β-catenin in organoids treated with/without FL4; n = 3. * *p* < 0.05.

The porcine intestinal organoids were successfully recovered and passaged two times. Shown in Supplementary Figure S2, the organoids grew slowly and asynchronously when they were just resuscitated; after two passages, the organoids matured within five days with good budding and the growth rate was significantly accelerated (Figure 4A). To assess the positive effects of *L. fermentum* FL4 on the intestinal epithelium, the organoids and *L. fermentum* FL4 (10^6^ CFU) were co-cultured on the third day for 24 h. The surface area of intestinal basal-out organoids increased significantly (*p* = 0.049) when treated with *L. fermentum* FL4 (Figure 4B). The significant increase (*p* = 0.042) in Lgr5 mRNA expression indicated the *L. fermentum* FL4 treatment might stimulate intestinal epithelial proliferation (Figure 4D). Considering Wnt/β-catenin signals are essential in intestinal epithelium homeostasis, correlation genes were further examined. *L. fermentum* FL4 significantly upregulated the mRNA expression of Wnt3a (*p* = 0.047), and the protein level of β-catenin was significantly increased compared with the control (Figure 4F).

### 3.6. L. fermentum FL4 Alleviated the Intestinal Epithelial Damage Induced by ETEC K88 in Basal-Out Intestinal Organoids

**Figure 5:**
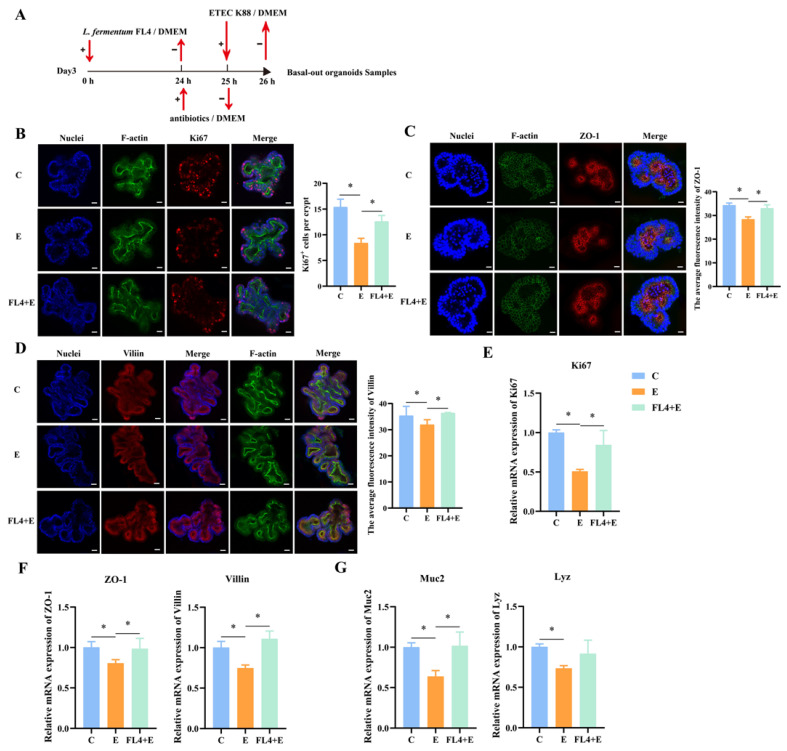
*L. fermentum* FL4 alleviated the intestinal epithelial damage induced by ETEC K88 in basal-out intestinal organoids. (**A**) Experimental procedure: basal-out organoids were treated with 10^6^ CFU/well *L. fermentum* FL4 at 0 h (Day 3) and then supplemented with antibiotics at 24 h. The organoids were further challenged with 10^6^ CFU/well ETEC K88 and the samples were collected at 26 hours. (**B**–**D**) Basal-out organoids were stained with Ki67, ZO-1, Villin (red). Nuclei were stained with 4′,6-diamidino-2-phenylindole (DAPI, blue), cytoskeletons were stained with F-actin (green). Scale bars, 20 μm. (**E**) RT-qPCR analysis of the Ki67 in organoids, n = 3. (**F**,**G**) RT-qPCR analysis of the ZO-1, Villin, Muc2 and lysozyme (Lyz) in organoids, n = 3. * *p* < 0.05.

**Figure 6:**
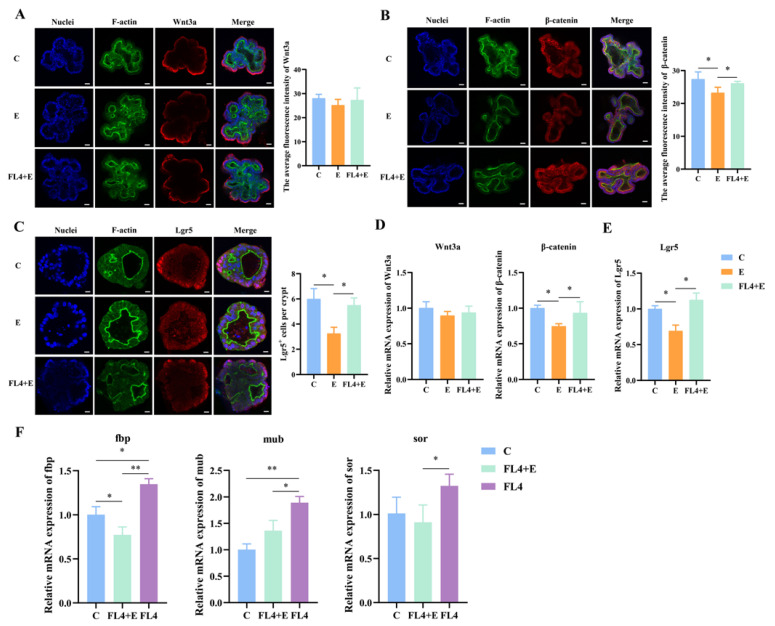
*L. fermentum* FL4 affected the Wnt/β-catenin pathway to alleviate the damage induced by ETEC K88. (**A**,**B**) Basal-out organoids were stained with Wnt3a and β-catenin (red). Nuclei were stained with 4′,6-diamidino-2-phenylindole (DAPI, blue), cytoskeletons were stained with F-actin (green). Scale bars, 20 μm. (**C**) Basal-out organoids were stained with Lgr5 (red). Nuclei were stained with 4′,6-diamidino-2-phenylindole (DAPI, blue), cytoskeletons were stained with F-actin (green). Scale bars, 20 μm. (**D**,**E**) RT-qPCR analysis of the Wnt3a, β-catenin and Lgr5 in organoids, n = 3. (**F**) Relative expression of adhesion marker genes fbp, mub, sor, n = 3. * *p* < 0.05, ** *p* < 0.01.

In addition to evaluating the stimulatory effects of *L. fermentum* under normal conditions, we also determined whether *L. fermentum* had a repair function on the damage caused by ETEC K88 in intestinal organoids (Figure 5A). Significant reduction (*p* = 0.012) of ZO-1 was observed in Figure 5C that indicated the mucosal barrier damage. Moreover, compared to the control, the villin and ZO-1 protein levels were decreased in organoids treated by ETEC K88 (Figure 5C,D). However, *L. fermentum* ameliorated the loss of ZO-1 to maintain the mucosal integrity (Figure 5F). Goblet cells and Paneth cells are secretory cells differentiated from stem cells in the intestine, and Mucin-2 (Muc2) and lysozyme (Lyz) are their respective markers. ETEC K88 stimulation significantly downregulated the mRNA expression of Muc2 (*p* = 0.015) and Lyz (*p* = 0.011) (Figure 5G). Moreover, the Ki67 mRNA levels (*p* = 0.011) and Ki67^+^ cell numbers were decreased (*p* = 0.036). Furthermore, *L. fermentum* reversed the reduced expression of Ki67 after ETEC K88 treatment (Figure 5B,E). Compared to the ETEC K88 alone-treated group, *L. fermentum* significantly enhanced mRNA and protein expression of β-catenin (*p* = 0.048). However, expression of wnt3a remained unchanged among three treatments (Figure 6A,B,D). The maintenance of ISCs was also further verified by the increase in the Lgr5^+^ cell number (*p* = 0.025) and Lgr5 mRNA expression levels (*p* = 0.012) in organoids treated with *L. fermentum* FL4 (Figure 6C,E).

We also analyzed the expression of adhesion genes such as fbp, mub and sor in *L. fermentum*; ETEC K88 stimulation significantly decreased fbp (*p* = 0.003), mub (*p* = 0.012) and sor (*p* = 0.026) expression (Figure 6F). The downregulation of adhesion genes may affect mitigation by *L. fermentum* of the intestinal epithelial damage in basal-out organoids because of less direct contact between *L. fermentum* and epithelial tips.

### 3.7. Establishment of Apical-Out Intestinal Organoids and Effects of L. fermentum FL4

**Figure 7:**
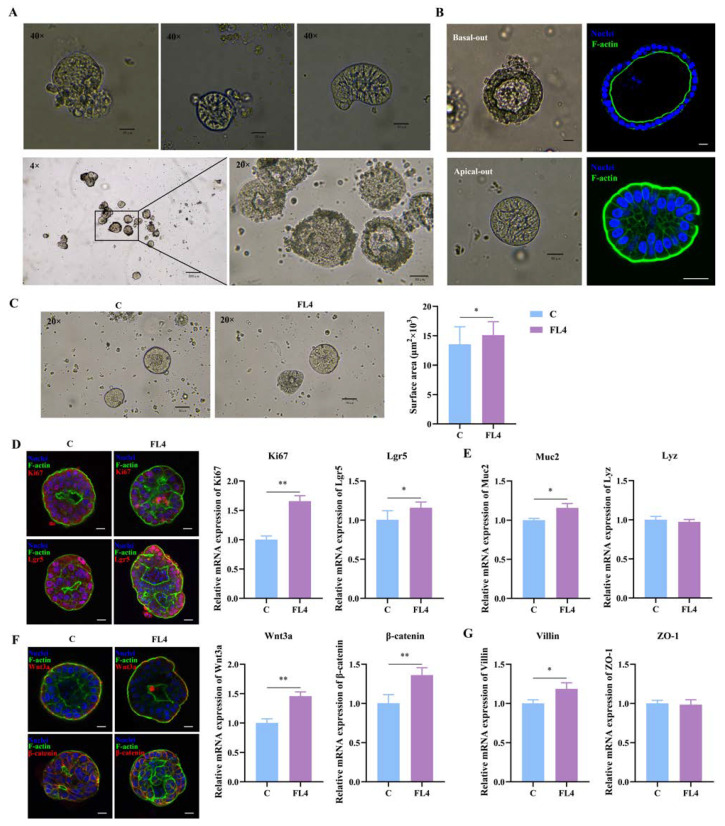
The establishment of apical-out intestinal organoids and the effects of *L. fermentum* FL4. (**A**) The procedure for polarity reversal of basal-out organoids to apical-out organoids and the shape of mature organoids. 4×, scale bars, 200 μm; 20×, scale bars, 50 μm; 40×, scale bars, 20 μm. (**B**) Contrast of organoid with basal-out polarity embedded in Matrigel or apical-out polarity in suspension depicted by micrographs (left) and confocal immunofluorescence images (right). Scale bars, 20 μm. (**C**) Organoids were treated with or without *L. fermentum* FL4 (10^6^ CFU/well) for 24 h. The surface area of organoids was calculated. 10×, scale bars, 200 μm; n = 6. (**D**) Confocal images of organoid staining with Nuclei (blue), F-actin (green), Ki67 and Lgr5 (red); scale bars, 20 μm. RT-qPCR analysis of the Ki67 and Lgr5 in organoids treated with/without FL4; n = 3. (**E**) RT-qPCR analysis of the Muc2, lysozyme (Lyz) in organoids treated with/without FL4; n = 3. (**F**) Confocal images of organoid staining with Wnt3a and β-catenin (red); scale bars, 20 μm. RT-qPCR analysis of the Wnt3a and β-catenin in organoids treated with/without FL4; n = 3. (**G**) RT-qPCR analysis of the villin and ZO-1 in organoids treated with/without FL4; n = 3. * *p* < 0.05, ** *p* < 0.01.

It is challenging to study the interaction between the epithelial apical surface and microbes in the intestinal lumen in conventional organoid models. The 3D spheroids were a closed system where outside substances had contact with the epithelial base, such as the nutrients and microbes added in the culture medium [29]. Julia et al. attempted to reverse the polarity of the organoids so that the apical surface turned outward, establishing the apical-out model [45]. After the suspension culture, observation under the microscope (Figure 7A) showed the morphology of organoids had changed. Organoids embedded in Matrigel had a clear lumen, while in the suspended organoids the edge of the columnar epithelial cells could be seen, often without a lumen (Figure 7B). Confocal immunofluorescence imaging of F-actin revealed that the epithelial apical surface turned outward successfully (Figure 7B).

We hypothesized that *L. fermentum* would stimulate the proliferation of the intestinal epithelium more efficiently in the apical-out model than in the basal-out model. The organoids and *L. fermentum* FL4 (10^6^ CFU) were co-cultured on the third day for 24 h, and the surface area of intestinal apical-out organoids increased significantly in *L. fermentum* FL4 treatment (Figure 7C). As expected, the treatment of *L. fermentum* resulted in the significant upregulation of mRNA expression of Ki67 (*p* = 0.004) and Lgr5 (*p* = 0.045) (Figure 7D). There was also an increase of villin (*p* = 0.025) and Muc2 (*p* = 0.044) at mRNA levels (Figure 7E,G). The Wnt/β-catenin pathway was activated in organoids treated by *L. fermentum* with significantly increasing mRNA expression of Wnt3a (*p* = 0.006), which was also confirmed by the obvious increase of β-catenin (*p* = 0.006) mRNA expression (Figure 7F). 

### 3.8. L. fermentum FL4 Repaired the ETEC K88-Induced Damage to the Intestinal Epithelium in Apical-Out Intestinal Organoids

**Figure 8:**
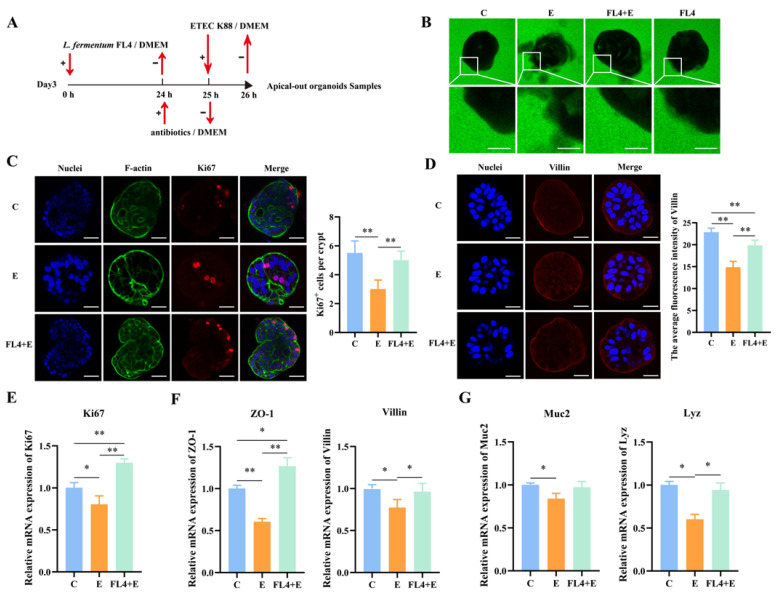
*L. fermentum* FL4 repaired the ETEC K88-induced damage to intestinal epithelium in apical-out intestinal organoids. (**A**) Experimental procedure: apical-out organoids were treated with 10^6^ CFU/well *L. fermentum* FL4 at 0 h (Day 3) and then supplemented with antibiotics at 24 h. The organoids were further challenged with 10^6^ CFU/well ETEC K88 and the samples were collected at 26 hours. (**B**) Apical-out organoids were imaged by using confocal fluorescence microscopy to observe epithelial barrier integrity in different treatments. Scale bars, 50 µm. (**C**,**D**) Apical-out organoids were stained with Ki67 and Villin (red). Nuclei were stained with 4′,6-diamidino-2-phenylindole (DAPI, blue), cytoskeletons were stained with F-actin (green). Scale bars, 20 μm. (**E**) RT-qPCR analysis of the Ki67 in organoids, n = 3. (**F**,**G**) The mRNA expression of the ZO-1, Villin, Muc2 and lysozyme (Lyz) in organoids, n = 3. * *p* < 0.05, ** *p* < 0.01.

To evaluate whether apical-out intestinal organoids can be applied as effective models for probing infection, ETEC K88 is used as a bacterial pathogen to invade intestinal epithelial cells (Figure 8A). The dextran diffusion assay was used, and we found that infection of apical-out organoids with ETEC K88 resulted in compromised barrier integrity. Imaged under the fluorescence confocal microscope, the organoids in the control completely excluded the FITC-dextrans, while in those treated with ETEC K88 the FITC-dextrans diffused into the inside of the 3D spheroids (Figure 8B). The decreased mRNA expression of ZO-1 (*p* = 0.001) and protein expression of villin further verified tight junctions disruption of barrier integrity (Figure 8D,F). 

**Figure 9:**
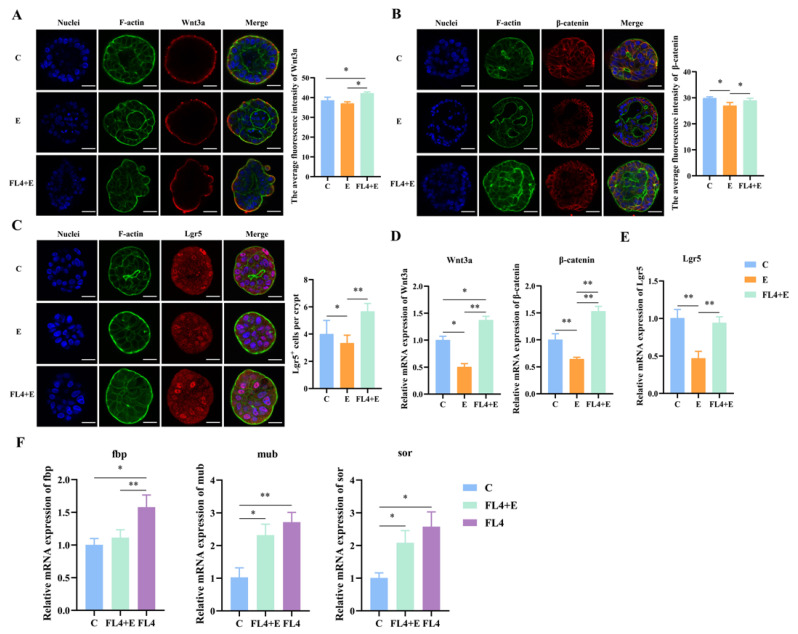
*L. fermentum* FL4 enhanced ISC regeneration by moderately activating the Wnt/β-catenin pathway. (**A**,**B**) Apical-out organoids were stained with Wnt3a and β-catenin (red). Nuclei were stained with 4′,6-diamidino-2-phenylindole (DAPI, blue), cytoskeletons were stained with F-actin (green). Scale bars, 20 μm. (**C**) Basal-out organoids were stained with Lgr5 (red). Nuclei were stained with 4′,6-diamidino-2-phenylindole (DAPI, blue), cytoskeletons were stained with F-actin (green). Scale bars, 20 μm. (**D**,**E**) RT-qPCR analysis of the Wnt3a, β-catenin and Lgr5 in organoids, n = 3. (**F**) Relative expression of adhesion marker genes fbp, mub, sor, n = 3. * *p* < 0.05, ** *p* < 0.01.

Additionally, we explored whether the *L. fermentum* could more effectively alleviate the ETEC K88-induced damage to the intestinal epithelium in apical-out intestinal organoids and maintain the integrity of the intestinal epithelial barrier. Pretreated by *L. fermentum*, the expression levels of ZO-1 (*p* = 0.001) and villin (*p* = 0.022) were significantly increased compared to the control and the ETEC K88 alone-treatment (Figure 8F). Moreover, *L. fermentum* prominently increased Ki67 (*p* = 0.001) as well as Ki67^+^ cells (Figure 8C,E). Compared to the organoids treated with ETEC K88 alone, *L. fermentum* significantly upregulated Wnt3a expression (*p* = 0.003), which was consistent with the significant increase (*p* = 0.005) in β-catenin (Figure 9A,B,D). These results indicated that *L. fermentum* activated the Wnt/β-catenin pathways to maintain inhibition caused by ETEC K88 and then enhanced the proliferative and differentiative ability of ISCs to repair the damaged epithelia. The significant increase in Lgr5 expression (*p* = 0.001) and Lgr5^+^ cell number (Figure 9C,D) in organoids treated with *L. fermentum* FL4 also proved this. ETEC K88 markedly decreased lysozyme expression (*p* = 0.041) compared with the control, indicating Paneth cell dysfunction. However, *L. fermentum* FL4 reversed the reduced expression of lysozyme (Figure 8C). 

Expression of adhesion genes such as fbp (*p* = 0.004), mub (*p* = 0.004) and sor (*p* = 0.026) was significantly increased both in groups of *L. fermentum* alone-treated and *L. fermentum* pretreated with ETEC K88 attack (Figure 9F), indicating that the adhesion of *L. fermentum* was less affected by ETEC K88 invasion. We speculated that the particular structure of apical-out intestinal organoids provides the potential to enhance adhesion of probiotics to intestinal epithelial cells.

### 3.9. L. fermentum (FL4) Modulated ETEC K88-Induced Inflammatory Responses in the Intestinal Epithelium

**Figure 10:**
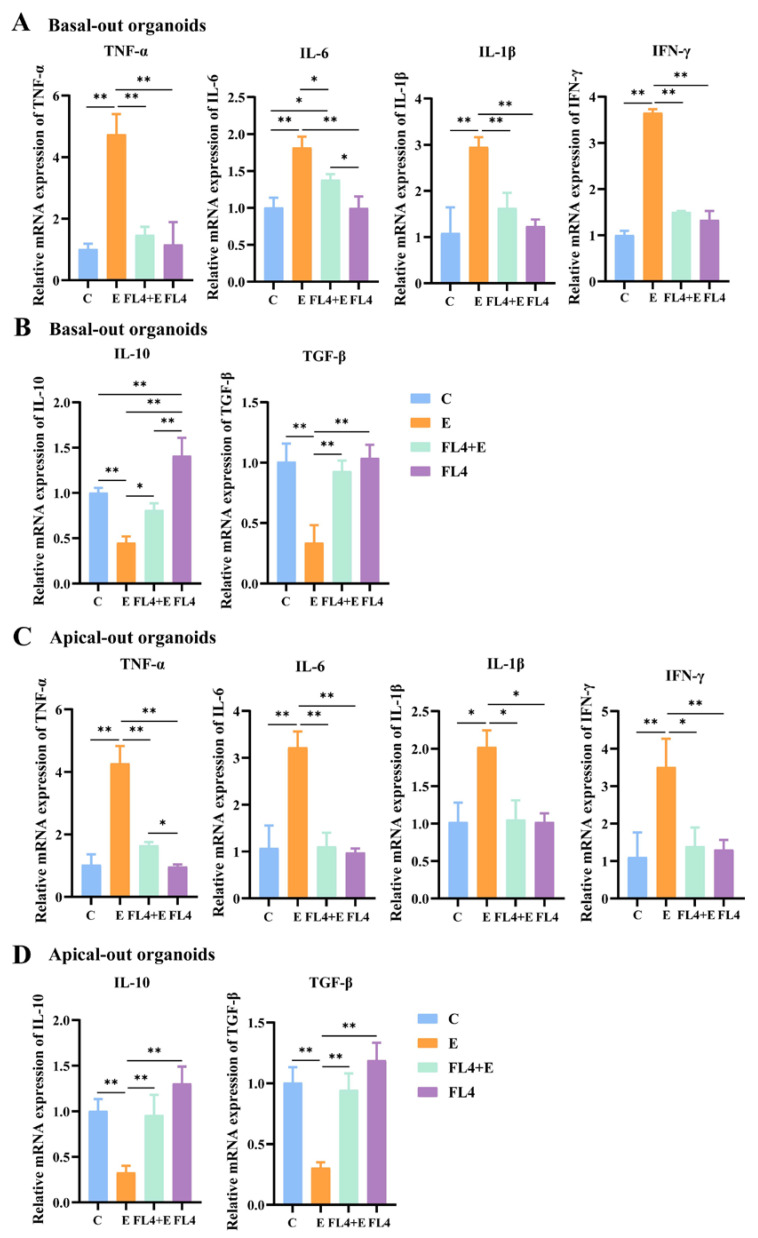
*L. fermentum* (FL4) modulated ETEC K88-induced inflammatory responses. (**A**) Expression of pro-inflammatory cytokines TNF-α, IL-6, IL-1β and IFN-γ treated with/without ETEC K88 or FL4 in basal-out organoids. (**B**) Expression of anti-inflammatory cytokines IL-10 and TGF-β treated with/without ETEC K88 or FL4 in basal-out organoids. (**C**) Expression of pro-inflammatory cytokines TNF-α, IL-6, IL-1β and IFN-γ treated with/without ETEC K88 or FL4 in apical-out organoids. (**D**) Expression of anti-inflammatory cytokines IL-10 and TGF-β treated with/without ETEC K88 or FL4 in apical-out organoids. n = 3. * *p* < 0.05, ** *p* < 0.01.

To verify the hypothesis that *L. fermentum* mitigates pathogen-induced inflammatory responses by modulating cytokines, intestinal epithelial cells were challenged with ETEC K88. Exposed to ETEC K88, the basal-out organoids dramatically enhanced the transcription of pro-inflammatory cytokines TNF-α, IL-6, IL-1β and IFN-γ, individually compared to the control as showed in Figure 8A. Preprocessing with *L. fermentum* alleviated the ETEC K88-induced inflammatory responses by the significant reduction of TNF-α, IL-1β and IFN-γ expression. Additionally, the transcriptional expression of anti-inflammatory markers IL-10 and TGF-β were significantly reduced after attack by ETEC K88. Furthermore, organoid interaction with *L. fermentum* tremendously increased the mRNA expression of regulatory cytokines (IL-10 and TGF-β) in comparison to ETEC K88-attacked organoids (Figure 10B). It is worth mentioning that the treatment with *L. fermentum* significantly increased IL-10 expression. Similar results were observed in experiments with apical-out organoids. ETEC K88-mediated higher pro-inflammatory cytokine (TNF-α, IL-6, IL-1β and IFN-γ) levels were significantly reduced upon incubation with *L. fermentum* (Figure 10C). Lower transcription of IL-10 and TGF-β was noticed when ETEC K88 stimulated the intestinal epithelial cells but markedly enhanced when *L. fermentum* was co-cultured in advance (Figure 10D).

## 4. Discussion

The intestinal microbiota of native pigs is rich and diverse; through multi-omics technology, it has been found that the abundance of Firmicutes is higher in native pigs than in introduced pigs [46]. Therefore, dominant strains such as *Lactobacillus* and *Bacillus* could be specifically excavated to study their effects on pigs. Zhang et al. [47] obtained *Lactobacillus* from the intestine of Rongchang pigs, which could be used to develop probiotics and promote stress resistance. Wang [48] isolated *Bacillus subtilis* MZ-01 from Min pig feces. It has a good effect on reducing the diarrhea of piglets and inhibiting the expression of inflammatory factors. We found that the abundance of *Lactobacillus* and *Limosilactobacillus fermentum* was significantly higher in Tunchang pigs (TC) than in Duroc × Landrace × Yorkshire (DLY) by metagenomic sequencing. In this study, four *L. fermentum* strains were isolated from Tunchang piglet feces, then the probiotic properties were tested under the FAO/WHO criteria for probiotics (2002) [49]. 

One desirable property of potential probiotics is surviving under GIT conditions (tolerance to low pH, pepsin, pancreatin and bile salts). The low pH in the stomach is a challenge for bacterial survival, especially since the pH at 2.0 and below often significantly decreases their viability. Our study showed that four *L. fermentum* strains survived in low pH, artificial gastrointestinal juice within 3 h. Different results in survival rates of *L. fermentum* were relative to different sources [9,50]. 

Another essential property in probiotics is the ability to adhere to intestinal mucosa. Hydrophobicity and auto-aggregation are used as primary markers for selecting probiotics, which affect adhesion mechanisms and pathogen colonization [51]. Four strains of *L. fermentum* showed high auto-aggregation, ranging from 93.12% to 94.47%. Aggregation appeared to be specific to each strain and may vary in the same species [52]. Additionally, the results of cell surface hydrophobicity ranged from 53.90% to 90.16% when the non-polar solvent was toluene. 

Antioxidant capacity is one of the main characteristics of LAB. The antioxidant capacity of the fermentation supernatant was always much stronger than the strain itself, which was consistent with Shen et al. [53]. This may be due to the metabolic components of the fermented strain, such as extracellular polysaccharides [54] and organic acids [55], having strong antioxidant capacity [56]. Additionally, the fermentation supernatant of FL4 showed the best inhibitory effect on ETEC K88. Research has revealed that the mechanism of killing Gram-negative bacteria is based on the production of organic acids, hydrogen peroxide and hydroxy fatty acids [57]. Low pH could help the passage of organic acids through the membrane to the intracellular environment. 

Organoids have been considered to be effective models for studying the interplay between host and bacteria due to their close relationship to tissues/organs. The pathogenesis of porcine epidemic diarrhea virus (PEDV) [58], *Salmonella* [59] has been explored in intestinal organoid models. Also, studies of the regulatory effect of microbiota (*L. reuteri* [60], *Akkermansia muciniphila* [61]) on ISCs and the interaction with them have been reported. ETEC K88 is the principal microorganism responsible for bacterial diarrhea in pigs, which commonly colonizes in the jejunum and releases enterotoxins to impair the intestinal barrier function and trigger an inflammatory reaction [62]. FL4 reduced the damage induced by ETEC K88; The increase of villin and ZO-1 mRNA expression as well as the number of Ki67^+^ and Lgr5^+^ cells [60] indicated FL4 alleviated intestinal epithelial damage. Wu et al. [60] reported that *L. reuteri* D8 maintained the activation of the Wnt/β-catenin pathway to guarantee the proliferation of intestinal epithelia. From results in our study, FL4 significantly enhanced expression of β-catenin. We speculate that epithelial cell organoids with basolateral surfaces out reduced the adhesion of *L. fermentum*. Several cell surface-associated proteins associated with mucus and enterocytes have been identified by genomic approaches, namely *mub*, *fbp*, S-layer proteins, LTA and exopolysaccharides [63]. ETEC K88 stimulation significantly reduced the expression of fbp, mub and sor.

Co et al. [45] established apical-out polarity of organoids that preserve epithelial integrity, maintain secretory and absorptive functions and allow regulated differentiation. After co-culture, the mRNA level of villin, Muc2, Ki67 and Lgr5 significantly increased, and the Wnt/β-catenin pathway was activated. Moreover, FL4 reduced damages induced by ETEC K88 to the intestinal barrier, reflected by the significant increase in ZO-1, villin, Lyz, Ki67, Lgr5, Ki67^+^ and Lgr5^+^ cells. The Wnt/β-catenin pathway is crucial for the maintenance of intestinal crypt proliferation [64,65]. *L. fermentum* FL4 activated the Wnt/β-catenin pathway with increased expression of Wnt3 and β-catenin to support intestinal epithelial proliferation and differentiation. The upregulation of adhesion genes *fbp*, *mub* and *sor* confirm the enhanced adhesion of *L. fermentum* to apical-out organoids. In summary, *L. fermentum* FL4 adhered to the apical surfaces more efficiently than to basolateral surfaces, which accelerated the Wnt/β-catenin signal activation to protect the mucosal barrier integrity and repair the ETEC K88-induced damage.

Cytokine production is considered to be an important indicator in response to ETEC K88. ETEC K88 significantly increased the expression of TNF-α, IL-1β and IFN-γ, while FL4 remarkably reduced these effects. Duary et al. [66] also reported lower levels of TNF-α, IL-8 and IFN-γ in *L. plantarum*+LPS treatment. These results suggest that FL4 intervention could control ETEC K88-induced inflammatory responses. Exposed to *L. fermentum*, FL4 enhanced the expression of TGF-β and IL-10 in organoids challenged by ETEC K88. *Lactobacillus* amplified TGF-β production through NF-κB signaling and inhibited inflammatory responses [67], which confirmed the anti-inflammatory properties of *Lactobacillus*.

## 5. Conclusions

In conclusion, we obtained four *L. fermentum* strains isolated from feces of healthy Tunchang piglets, and they exhibited probiotic properties, including resistance to gastrointestinal conditions, adhesion ability, antimicrobial activity against ETEC K88 and antioxidant capacity. Further in vitro experiments in basal-out and apical-out organoids demonstrated that *L. fermentum* adhered to the apical surfaces more efficiently than to the basolateral surfaces, had the ability to activate the Wnt/β-catenin pathway to protect the mucosal barrier integrity, stimulated the proliferation and differentiation of the intestinal epithelium, and repaired the ETEC K88-induced damage. Moreover, *L. fermentum* inhibited inflammatory responses induced by ETEC K88 through the reduced expression of pro-inflammatory cytokines (TNF-α, IL-1β and IFN-γ) and higher levels of anti-inflammatory cytokines (TGF-β and IL-10). Further in vivo experiments and clinical studies of pigs are required to ascertain the safety and efficacy of Tunchang pig-derived probiotics.

## Figures and Tables

**Table 1:** Primers used for qRT-PCR.

Gene	Primer	Reference
Lyz	Forward GGTCTATGATCGGTGCGAGT	Gonzalez et al., 2013 [35]
	Reverse AACTGCTTTGGGTGTCTTGC	
Muc2	Forward GGCTGCTCATTGAGAGGAGT	Gonzalez et al., 2013 [35]
	Reverse ATGTTCCCGAACTCCAAGG	
Villin	Forward ACGTGTCTGACTCCGAGGGAAAGGT	Yin et al., 2022 [36]
	Reverse ACTGCTTCGCTTTGATAAAGTTCAG	
ZO-1	Forward CCGCCTCCTGAGTTTGATAG	He et al., 2020 [37]
	Reverse CAGCTTTAGGCACTGTGCTG	
Ki67	Forward AGTCTGTAAGGAAAGCCACCC	Claire et al., 2018 [38]
	Reverse ACAAAGCCCAAGCAGACAGG	
Lgr5	Forward CCTTGGCCCTGAACAAAATA	Gonzalez et al., 2013 [35]
	Reverse ATTTCTTTCCCAGGGAGTGG	
Wnt3a	Forward GCGACTTCCTCAAGGACAAG	Gonzalez et al., 2013 [35]
	Reverse GGTCACGTGTACCGAAGGAT	
β-Catenin	Forward GGCTCTTGTGCGTACTGTCCTTC	Han et al., 2021 [39]
	Reverse TTCCTGGTGTCGGCTGGTCAG	
GAPDH	Forward ATCCTGGGCTACACTGAGGAC	Gonzalez et al., 2013 [35]
	Reverse AAGTGGTCGTTGAGGGCAATG	
*fbp*	Forward GACAAGCGGAAGGTCAAGAA	Archer et al., 2018 [40]
	Reverse GTCGTAGAAGTTGGGCAGTT	
*mub*	Forward GCAAGGACCAGTCAGAGTTA	Archer et al., 2018 [40]
	Reverse GCCCAAAGAACCACAGGTAG	
*sor*	Forward GTGATCATTTTGGGGCTTGC	Archer et al., 2018 [40]
	Reverse CGCTGCTGAAATCGTAACTG	
*pheS*	Forward GACCACGATTCCTTCAACCT	Archer et al., 2018 [40]
	Reverse TGGAACAACACGACTTCTCC	
TNF-α	Forward CGCTCTTCTGCCTACTGCACTT	Geng et al., 2018 [41]
	Reverse CGGCTTTGACATTGGCTACAA	
IL-6	Forward GCCTTCAGTCCAGTCGCCTTC	Geng et al., 2018 [41]
	Reverse GTGGCATCACCTTTGGCATCTTC	
IL-1β	Forward GCCAGTCTACATTGCTCATGTTTCT	Geng et al., 2018 [41]
	Reverse GTTGTCACCATTGTTAGCCATCAC	
IFN-γ	Forward CAGAGCCAAATTGTCTCCTTCTAC	Geng et al., 2018 [41]
	Reverse GTCATTCAGTTTCCCAGAGCTACCA	
IL-10	Forward GACCAGATGGGCGACTTGTTG	Geng et al., 2018 [41]
	Reverse GGGAGTTCACGTGCTCCTTGAT	
TGF-β	Forward GAAGCGCATCGAGGCCATTC	Geng et al., 2018 [41]
	Reverse GGCTCCGGTTCGACACTTTC	

**Table 2:** Survival rate (%) of *Limosilactobacillus fermentum* strains under simulated gastrointestinal tract conditions.

Strains	pH = 2	pH = 3	Simulated Gastric Juices	Simulated Intestinal Juices
FL1	57.39 + 0.76 ^b^	95.65 + 0.33 ^a^	55.91 + 0.83 ^b^	65.03 + 2.73 ^b^
FL2	61.40 + 1.51 ^a^	87.61 + 0.47 ^b^	58.15 + 0.84 ^ab^	67.38 + 2.68 ^b^
FL3	52.18 + 1.34 ^c^	97.76 + 0.90 ^a^	57.90 + 0.43 ^b^	70.83 + 0.71 ^ab^
FL4	65.63 + 0.59 ^a^	90.02 + 0.78 ^b^	59.95 + 0.26 ^a^	73.57 + 0.57 ^a^

Data were expressed as the mean ± SD (n = 3). Different lowercase letters (a, b, c) in the same column indicated significant differences, *p* < 0.05.

## Data Availability

The whole genome sequence of the four tested strains has been deposited at GeneBank ID SUB12926851, with accession numbers OQ552786 to OQ552789.

## Supplementary Materials

The following supporting information can be downloaded at: https://www.mdpi.com/article/10.3390/microorganisms11041055/s1, Supplementary Figure S1: Isolation and identification of Limosilactobacillus fermentum strains. Supplementary Figure S2: Culture of porcine intestinal organoids.

## References

[1] Dowarah R., Verma A.K., Agarwal N., Singh P. (2018). Efficacy of species-specific probiotic *Pediococcus acidilactici* FT28 on blood biochemical profile, carcass traits and physicochemical properties of meat in fattening pigs. Res. Vet. Sci..

[2] Zamojska D., Nowak A., Nowak I., Macierzyńska-Piotrowska E. (2021). Probiotics and Postbiotics as Substitutes of Antibiotics in Farm Animals: A Review. Animals.

[3] Yang F., Hou C., Zeng X., Qiao S. (2015). The use of lactic Acid bacteria as a probiotic in Swine diets. Pathogens.

[4] Bintsis T. (2018). Lactic acid bacteria: Their applications in foods. J. Bacteriol. Mycol..

[5] Huang J., Zhang W., Hu Z., Liu Z., Du T., Dai Y., Xiong T. (2020). Isolation, characterization and selection of potential probiotic lactic acid bacteria from feces of wild boar, native pig and commercial pig. Livest. Sci..

[6] Wang J., Ji H., Wang S., Liu H., Zhang W., Zhang D., Wang Y. (2018). Probiotic *Lactobacillus plantarum* Promotes Intestinal Barrier Function by Strengthening the Epithelium and Modulating Gut Microbiota. Front. Microbiol..

[7] Chiang M.L., Chen H.C., Chen K.N., Lin Y.C., Lin Y.T. (2015). Optimizing production of two potential probiotic Lactobacilli strains isolated from piglet feces as feed additives for weaned piglets. Asian Australas. J. Anim..

[8] Lim S.M., Lee N.K., Kim K.T., Paik H.D. (2020). Probiotic *Lactobacillus fermentum* KU200060 isolated from watery kimchi and its application in probiotic yogurt for oral health. Microb. Pathog..

[9] De Souza B.M.S., Borgonovi T.F., Casarotti S.N., Todorov S.D., Penna A.L.B. (2019). *Lactobacillus casei* and *Lactobacillus fermentum* Strains Isolated from Mozzarella Cheese: Probiotic Potential, Safety, Acidifying Kinetic Parameters and Viability under Gastrointestinal Tract Conditions. Probiotics Antimicrob. Proteins.

[10] Coton M., Berthier F., Coton E. (2008). Rapid identification of the three major species of dairy obligate heterofermenters *Lactobacillus brevis*, *Lactobacillus fermentum* and *Lactobacillus parabuchneri* by species-specific duplex PCR. FEMS Microbiol. Lett..

[11] Martín R., Langa S., Reviriego C., Jimínez E., Marín M.L., Xaus J., Fernández L., Rodríguez J.M. (2003). Human milk is a source of lactic acid bacteria for the infant gut. J. Pediatr..

[12] Poluektova E.U., Yunes R.A., Epiphanova M.V., Orlova V.S., Danilenko V.N. (2017). The *Lactobacillus rhamnosus* and *Lactobacillus fermentum* strains from human biotopes characterized with MLST and toxin-antitoxin gene polymorphism. Arch. Microbiol..

[13] Zhao Y., Yu L., Tian F., Zhao J., Zhang H., Chen W., Xue Y., Zhai Q. (2022). Environment-Related Genes Analysis of *Limosilactobacillus fermentum* Isolated from Food and Human Gut: Genetic Diversity and Adaption Evolution. Foods.

[14] Jang Y.J., Kim W.K., Han D.H., Lee K., Ko G. (2019). *Lactobacillus fermentum* species ameliorate dextran sulfate sodium-induced colitis by regulating the immune response and altering gut microbiota. Gut Microbes.

[15] Lin W., Yu B., Jang S., Tsen H. (2007). Different probiotic properties for *Lactobacillus fermentum* strains isolated from swine and poultry. Anaerobe.

[16] Mu J., Tan F., Zhou X., Zhao X. (2020). *Lactobacillus fermentum* CQPC06 in naturally fermented pickles prevents non-alcoholic fatty liver disease by stabilizing the gut–liver axis in mice. Food Funct..

[17] Liu J., Chen X., Zhou X., Yi R., Yang Z., Zhao X. (2021). *Lactobacillus fermentum* ZS09 Mediates Epithelial–Mesenchymal Transition (EMT) by Regulating the Transcriptional Activity of the Wnt/β-Catenin Signalling Pathway to Inhibit Colon Cancer Activity. J. Inflamm. Res..

[18] Galdeano C.M., Cazorla S.I., Dumit J.M.L., Vélez E., Perdigón G. (2019). Beneficial effects of probiotic consumption on the immune system. Ann. Nutr. Metab..

[19] Dargahi N., Matsoukas J., Apostolopoulos V. (2020). *Streptococcus thermophilus* ST285 alters pro-inflammatory to anti-inflammatory cytokine secretion against multiple sclerosis peptide in mice. Brain Sci..

[20] Villena J., Kitazawa H. (2014). Modulation of Intestinal TLR4-Inflammatory Signaling Pathways by Probiotic Microorganisms: Lessons Learned from *Lactobacillus jensenii* TL2937. Front Immunol..

[21] Sharma R., Kapila R., Kapasiya M., Saliganti V., Dass G., Kapila S. (2014). Dietary supplementation of milk fermented with probiotic *Lactobacillus fermentum* enhances systemic immune response and antioxidant capacity in aging mice. Nutr. Res..

[22] Bhat M.I., Kapila S., Kapila R. (2020). *Lactobacillus fermentum* (MTCC-5898) supplementation renders prophylactic action against Escherichia coli impaired intestinal barrier function through tight junction modulation. LWT-Food Sci. Technol..

[23] Chen C., Lai C., Huang H., Huang W., Toh H.S., Weng T., Chuang Y., Lu Y., Tang H. (2019). Antimicrobial activity of *Lactobacillus* species against carbapenem-resistant enterobacteriaceae. Front. Microbiol..

[24] Behera S.S., Ray R.C., Zdolec N. (2018). *Lactobacillus plantarum* with functional properties: An approach to increase safety and shelf-life offermented foods. Biomed. Res. Int..

[25] Gupta T., Kaur H., Kapila S., Kapila R. (2021). *Lactobacillus fermentum* (MTCC-5898) alleviates Escherichia coli-induced inflammatory responses in intestinal epithelial cells by modulating immune genes and NF-κB signalling. J. Appl. Microbiol..

[26] Rossi G., Manfrin A., Lutolf M.P. (2018). Progress and potential in organoid research. Nat. Rev. Genet..

[27] Gjorevski N., Sachs N., Manfrin A., Giger S., Bragina M.E., Ordóñez-Morán P., Clevers H., Lutolf M.P. (2016). Designer matrices for intestinal stem cell and organoid culture. Nature.

[28] Barker N., Huch M., Kujala P., van de Wetering M., Snippert H.J., van Es J.H., Sato T., Stange D.E., Begthel H., van den Born M. (2010). Lgr5(+ve) stem cells drive self-renewal in the stomach and build long-lived gastric units in vitro. Cell Stem Cell.

[29] Sato T., Vries R.G., Snippert H.J., van de Wetering M., Barker N., Stange D.E., van Es J.H., Abo A., Kujala P., Peters P.J. (2009). Single Lgr5 stem cells build crypt-villus structures in vitro without a mesenchymal niche. Nature.

[30] Sornsenee P., Singkhamanan K., Sangkhathat S., Saengsuwan P., Romyasamit C. (2021). Probiotic Properties of *Lactobacillus* Species Isolated from Fermented Palm Sap in Thailand. Probiotics Antimicrob. Proteins.

[31] Vlkova E., Rada V., Smehilova M., Killer J. (2008). Auto-aggregation and co-aggregation ability in bifidobacteria and clostridia. Folia Microbiol..

[32] Stepanovic S., Vukovic D., Dakic I., Savic B., Svabic-Vlahovic M. (2000). A modified microtiter plate test for quantification of staphylococcal biofilm formation. J. Microbiol. Methods.

[33] Co J.Y., Margalef-Català M., Monack D.M., Amieva M.R. (2021). Controlling the polarity of human gastrointestinal organoids to investigate epithelial biology and infectious diseases. Nat. Protoc..

[34] Falah F., Vasiee A., Behbahani B.A., Yazdi F.T., Moradi S., Mortazavi S.A., Roshanak S. (2019). Evaluation of adherence and anti-infective properties of probiotic *Lactobacillus fermentum* strain 4–17 against Escherichia coli causing urinary tract infection in humans. Microb. Pathog..

[35] Gonzalez L.M., Williamson I., Piedrahita J.A., Blikslager A.T., Magness S.T. (2013). Cell lineage identification and stem cell culture in a porcine model for the study of intestinal epithelial regeneration. PLoS ONE.

[36] Yin L., Li J., Zhang Y., Yang Q., Yang C., Yi Z., Yin Y., Wang Q., Li J., Ding N. (2022). Changes in progenitors and differentiated epithelial cells of neonatal piglets. Anim. Nutr..

[37] He Y., Jinno C., Kim K., Wu Z., Tan B., Li X., Whelan R., Liu Y. (2020). Dietary *Bacillus* spp. enhanced growth and disease resistance of weaned pigs by modulating intestinal microbiota and systemic immunity. J. Anim. Sci. Biotechnol..

[38] Stenhouse C., Hogg C.O., Ashworth C.J. (2018). Associations between fetal size, sex and both proliferation and apoptosis at the porcine feto-maternal interface. Placenta.

[39] Han Q., Liu H., Zhang R., Yang X., Bao J., Xing H. (2021). Selenomethionine protects against ammonia-induced apoptosis through inhibition of endoplasmic reticulum stress in pig kidneys. Ecotoxicol. Environ. Saf..

[40] Archer A., Kurrey N., Halami P. (2018). In vitro adhesion and anti-inflammatory properties of native *Lactobacillus fermentum* and *Lactobacillus delbrueckii* spp.. J. Appl. Microbiol..

[41] Geng S., Cheng S., Li Y., Wen Z., Ma X., Jiang X., Wang Y., Han X. (2018). Faecal Microbiota Transplantation Reduces Susceptibility to Epithelial Injury and Modulates Tryptophan Metabolism of the Microbial Community in a Piglet Model. J. Crohns Colitis.

[42] Sánchez-Ortiz A.C., Luna-González A., Campa-Córdova A.I., Escamilla-Montes R., Flores-Miranda M.D.C., Mazón-Suástegui J.M. (2015). Isolation and characterization of potential probiotic bacteria from pustulose ark (*Anadara tuberculosa*) suitable for shrimp farming. Lat. Am. J. Aquat. Res..

[43] Koss B., Suskovic J., Vukovic S., Simpraga M., Frece J., Matosic S. (2003). Adhesion and aggregation ability of probiotic strain *Limosilactobacillus acidophilus* M92. J. Ind. Microbiol. Biotechnol..

[44] Fessard A., Bourdon E., Payet B., Remize F. (2016). Identification, stress tolerance and antioxidant activity of lactic acid bacteria isolated from tropically-grown fruits and leaves. Can. J. Microbiol..

[45] Co J.Y., Margalef-Català M., Li X., Mah A.T., Kuo C.J., Monack D.M., Amieva M.R. (2019). Controlling Epithelial Polarity: A Human Enteroid Model for Host-Pathogen Interactions. Cell Rep..

[46] Ma N., Sun Y., Chen J., Qi Z., Liu C., Ma X. (2022). Micro-Coevolution of Genetics Rather Than Diet with Enterotype in Pigs. Front. Nutr..

[47] Zhang Y., Hu H., Xian X. (2016). Isolation and identification of lactic acid bacteria from intestinal tract of Rongchang pig. J. Southwest Univ..

[48] Wang X. (2022). Screening of Probiotic Strains and Analysis of Their Antibacterial Components from Min Pigs. Master’s Thesis.

[49] World Health Organization Guidelines for the Evaluation of Probiotics in Food (2002). https://www.who.int/foodsafety/fs_management/en/probiotic_guidelines.pdf.

[50] Kocabay S., Çetinkaya S. (2020). Probiotic Properties of a *Lactobacillus fermentum* Isolated from New-born Feces. J. Oleo Sci..

[51] Pereira G.V.M., Oliveira C.B., Magalhães Júnior A.I., Thomaz-Soccol V., Soccol C.R. (2018). How to select a probiotic? A review and update of methods and criteria. Biotechnol. Adv..

[52] Todorov S.D., Furtado D.N., Saad S.M., Tome E., Franco B.D. (2011). Potential beneficial properties of bacteriocin-producing lactic acid bacteria isolated from smoked salmon. J. Appl. Microbiol..

[53] Qian S., Shang N., Li P. (2011). In vitro and in vivo antioxidant activity of *Bifidobacterium animalis* 01 isolated from centenarians. Curr. Microbiol..

[54] Polak-Berecka M., Waśko A., Szwajgier D., Chomaz A. (2013). Bifidogenic and antioxidant activity of exopolysaccharides produced by *Lactobacillus rhamnosus* E/N cultivated on different carbon sources. Pol. J. Microbiol..

[55] Wu Y., Li S., Tao Y., Li D., Han Y., Show P.L., Wen G., Zhou J. (2021). Fermentation of blueberry and blackberry juices using *Lactobacillus plantarum*, *Streptococcus thermophilus* and *Bifidobacterium bifidum*: Growth of probiotics, metabolism of phenolics, antioxidant capacity in vitro and sensory evaluation. Food Chem..

[56] Vasquez E.C., Pereira T.M.C., Peotta V.A., Baldo M.P., Campos-Toimil M. (2019). Probiotics as beneficial dietary supplements to prevent and treat cardiovascular diseases: Uncovering their impact on oxidative stress. Oxidat. Med. Cell. Longev..

[57] Kivanc M., Yilmaz M., Çakir E. (2011). Isolation and identification of lactic acid bacteria from boza, and their microbial activity against several reporter strains. Turk. J. Biol..

[58] Li L., Fu F., Guo S., Wang H., He X., Xue M., Yin L., Feng L., Liu P. (2019). Porcine Intestinal Enteroids: A New Model for Studying Enteric Coronavirus Porcine Epidemic Diarrhea Virus Infection and the Host Innate Response. J. Virol..

[59] Yin Y., Zhou D. (2018). Organoid and Enteroid Modeling of Salmonella Infection. Front. Cell Infect. Microbiol..

[60] Wu H., Xie S., Miao J., Li Y., Wang Z., Wang M., Yu Q. (2020). *Lactobacillus* reuteri maintains intestinal epithelial regeneration and repairs damaged intestinal mucosa. Gut Microbes.

[61] Kim S., Shin Y.S., Kim T.Y., Kim Y., Lee Y.S., Lee S.H., Kim M.N., Eunju O., Kim K.S., Kweon M.N. (2021). Mucin degrader *Akkermansia muciniphila* accelerates intestinal stem cell-mediated epithelial development. Gut Microbes.

[62] Dubreuil J.D., Isaacson R.E., Schifferli D.M. (2016). Animal Enterotoxigenic *Escherichia coli*. EcoSal Plus.

[63] Buck B.L., Altermann E., Svingerud T., Klaenhammer T.R. (2005). Functional analysis of putative adhesion factors *in Lactobacillus acidophilus* NCFM. Appl. Environ. Microbiol..

[64] Farin H.F., Van Es J.H., Clevers H. (2012). Redundant sources of Wnt regulate intestinal stem cells and promote formation of Paneth cells. Gastroenterology.

[65] Clevers H., Loh K.M., Nusse R. (2014). Stem cell signaling. An integral program for tissue renewal and regeneration: Wnt signaling and stem cell control. Science.

[66] Duary R.K., Batish V.K., Grover S. (2014). Immunomodulatory activity of two potential probiotic strains in LPS-stimulated HT-29 cells. Genes Nutr..

[67] Bermudez-Brito M., Munoz-Quezada S., Gomez-Llorente C., Matencio E., Romero F., Gil A. (2015). *Lactobacillus paracasei* CNCM I-4034 and its culture supernatant modulate Salmonella-induced inflammation in a novel transwell co-culture of human intestinal-like dendritic and Caco-2 cells. BMC Microbiol..

