# Supplementary material for: Evaluation of Potential Probiotic Properties of Limosilactobacillus fermentum Derived from Piglet Feces and Influence on the Healthy and E. coli-Challenged Porcine Intestine
Source: Microorganisms. 2023 Apr 18;11(4):1055. doi: 10.3390/microorganisms11041055 (PMC10142273; doi:10.3390/microorganisms11041055)
Supplement: Supplementary file 1 [file microorganisms-11-01055-s001.zip › microorganisms-2294687-supplementary.pdf]

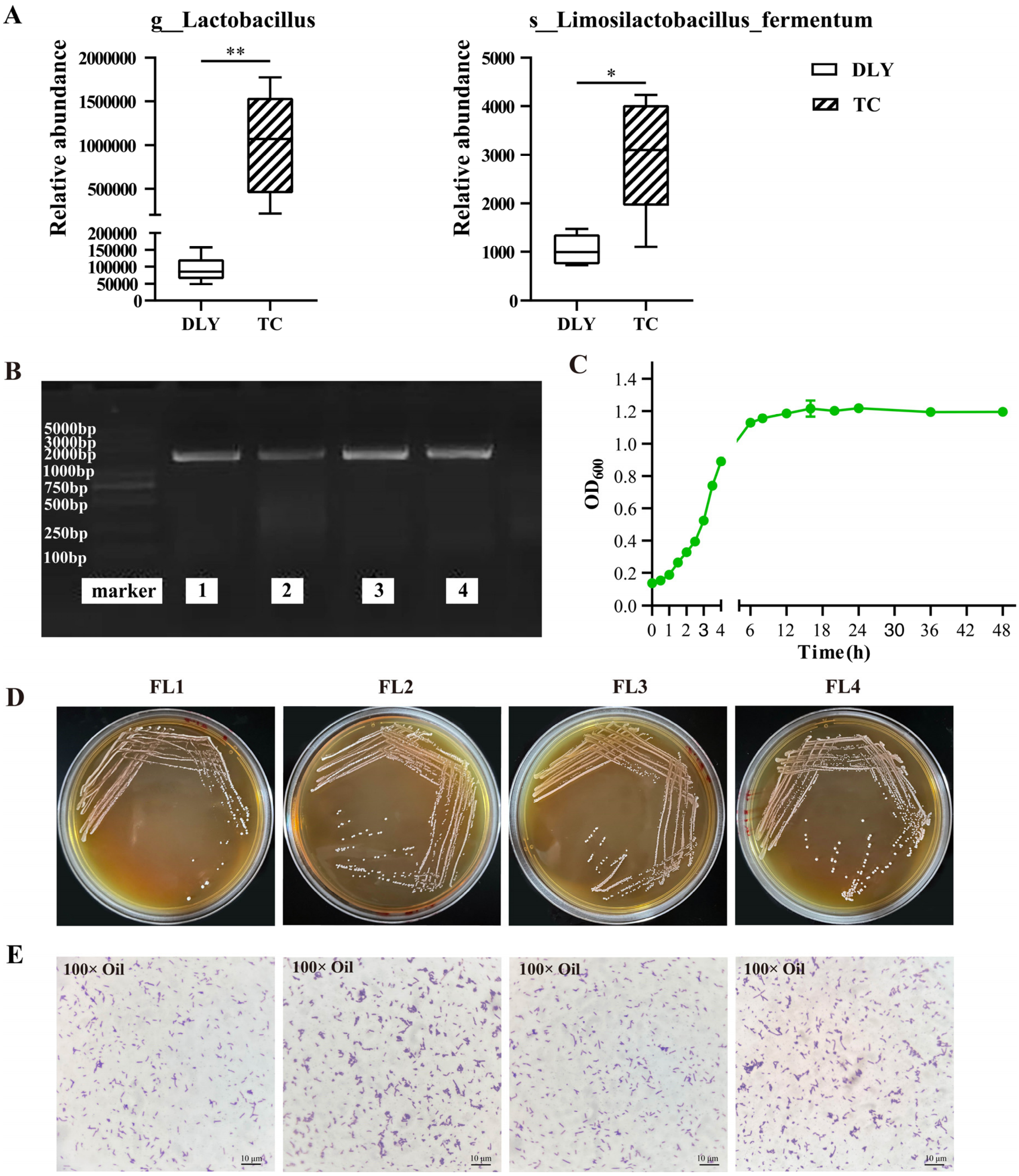

Supplementary Figure S1. Isolation and identification of *Limosilactobacillus fermentum* strains. (A) The relative abundance of *Lactobacillus* and *Limosilactobacillu fermentum* in Tunchang pigs (TC) and Duroc × Landrace × Yorkshire pigs (DLY). (B) Identification electrophoretogram of four bacteria. (C) The growth chart of *L. fermentum* FL4. (D) Colony morphology of isolated strains. (E) Cell morphology of strains under optical microscope, 100× oil immersion lens. Scale bars, 10 μm. * $p < 0.05$, ** $p < 0.01$.

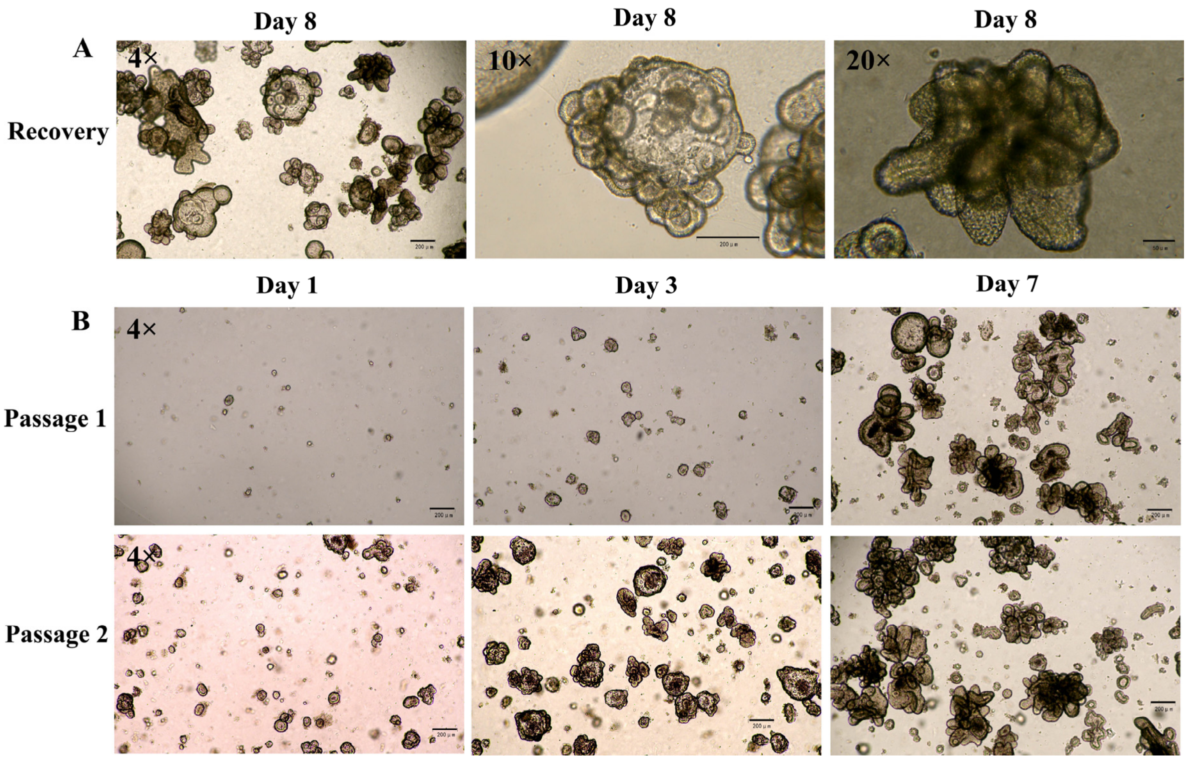

Supplementary Figure S2. Culture of porcine intestinal organoids. (A) Eighth day after frozen intestinal organoids were resuscitated. (B) The growth of intestinal organoids after passages, morphology individually at Day 1, Day 3 and Day 5.
